# A GeoHealth Call to Action: Moving Beyond Identifying Environmental Injustices to Co‐Creating Solutions

**DOI:** 10.1029/2022GH000706

**Published:** 2022-11-01

**Authors:** A. Hoffman‐Hall, M. E. Gorris, S. Anenberg, A. E. Bredder, J. K. Dhaliwal, M. A. Diaz, S. K. Fortner, B. G. McAdoo, D. Reano, R. C. Rehr, H. A. Roop, B. F. Zaitchik

**Affiliations:** ^1^ Environmental Studies Discipline Eckerd College St. Petersburg FL USA; ^2^ Los Alamos National Laboratory Los Alamos NM USA; ^3^ George Washington University Milken Institute School of Public Health Washington DC USA; ^4^ Department of Geographical Sciences University of Maryland College Park MD USA; ^5^ Earth and Planetary Sciences UC Santa Cruz Santa Cruz CA USA; ^6^ Woods Hole Oceanographic Institution Woods Hole MA USA; ^7^ Science Education Resource Center Carleton College Northfield MN USA; ^8^ Nicholas School of the Environment Duke University Durham NC USA; ^9^ School of Earth and Space Exploration Arizona State University Tempe AZ USA; ^10^ Maryland League of Conservation Voters Annapolis MD USA; ^11^ University of Minnesota Minneapolis MN USA; ^12^ University of Washington School of Public Health Seattle WA USA; ^13^ Department of Earth and Planetary Sciences Johns Hopkins University Baltimore MD USA

**Keywords:** environmental justice, community science, justice, health

## Abstract

As marginalized communities continue to bear disproportionate impacts from environmental hazards, we urgently call for researchers and institutions to elevate the principles of Environmental Justice. The American Geophysical Union (AGU) GeoHealth section supports members' engagement in health‐related community‐engaged and community‐led transdisciplinary research. We highlight intersectional research that provides examples and actions for both individuals and organizations on community science and trust building, removing barriers created by scientific agency priorities and career expectations, and opportunities in education and policy. Justice does not start or end at one meeting; this is ongoing work that is active, evolving, and an ethical responsibility of AGU's membership.

## Key Points

- We, members of American Geophysical Union GeoHealth, urgently call for researchers to incorporate and elevate the principles of Environmental Justice in their work
- We discuss justice‐oriented capacity‐building strategies like co‐creating research with communities, education, and restructuring funding mechanisms
- We provide individual‐ and organizational‐level guidance on converting science into action, dismantling barriers, and overcoming challenges

## 1 Introduction

Marginalized communities have historically, and are currently, bearing disproportionate impacts from environmental hazards and pollution. Multiple medical organizations have declared racism a public health crisis, including the American Public Health Association, American College of Emergency Physicians, and American Hospital Association (ACEP, 2020; AHA, 2021; APHA, 2022). With awareness and acknowledgment of the intersection of racism and social determinants of health (Franks et al., 2021), we call for GeoHealth scientists and practitioners to incorporate and elevate the principles of the well‐established field of Environmental Justice in their work. As defined by Dr. Robert D. Bullard, often referred to as The Father of Environmental Justice, “Environmental Justice embraces the principle that all people and communities are entitled to equal protection of environmental and public health laws and regulations” (Bullard, 1996). Now, more than ever, is the time to move from identifying, quantifying, and communicating environmental injustices (an area in which many of our colleagues have done and are doing important work), to more robust capacity building including co‐creating solutions and building the infrastructure and education needed for full collaboration to development of solutions to these injustices.

The emerging field of GeoHealth combines earth, environmental, and health sciences across disciplinary boundaries to identify societally relevant scientific questions, collaborate with frontline communities, and develop evidence needed for decisions at all scales (Barnard et al., 2021; Hayhow et al., 2021). Established in 2017, the GeoHealth section within the American Geophysical Union (AGU) has been increasingly active. GeoHealth research and topics have been increasingly addressed in journals and at the AGU Fall Meeting, especially in response to the COVID‐19 pandemic (Gorris et al., 2021). One of the core missions of the GeoHealth section is to support and enhance AGU members' engagement in community‐engaged and community‐led transdisciplinary research across all aspects of health. GeoHealth research has the ability to directly impact human well‐being, with a much quicker research‐to‐action timeline than many other fields of Geoscience, which has been well demonstrated in COVID‐19 research (Gorris et al., 2021). As such, it is important to ensure that this research‐to‐action pipeline remains a key priority of GeoHealth practitioners and scientists while being catalyzed and led by community priorities. The inclusion and elevation of community priorities is a key principle of the field of Environmental Justice. As has been shown in many scientific and research contexts, the inclusion of historically marginalized communities in geoscience research results in higher quality research and broad, critical, and timely societal impacts (Ballard & Huntsinger, 2006; Bonney et al., 2009; Dalbotten et al., 2017; Danielsen et al., 2014; Harris et al., 2021; Hoffman‐Hall et al., 2020; Moysey, 2011; Pierotti & Wildcat, 2000; Price & Lee, 2013; Ramirez‐Andreotta et al., 2014, 2016; Shirk et al., 2012; Smythe et al., 2010; Southern et al., 2022).

We advocate that Environmental Justice should have a permanent presence at all future AGU meetings, across all sections. We highlight intersectional GeoHealth and Environmental Justice work that provides examples and guidance on converting science into action, identify barriers to conducting this work, and offer suggestions to overcome challenges. Through this sample of work presented, we provide a framework for incorporating Environmental Justice in future AGU meetings and research, regardless of section membership or disciplinary field. Environmental Justice is constantly evolving. We hope that this work might also guide how other professional organizations incorporate robust plans for equity and justice.

## 2 Where We Are: AGU Fall Meeting 2021

During the 2021 Fall Meeting (FM21) of the AGU, Environmental Justice was a prominent theme across disciplinary sections, with the term “Justice” appearing in 10 session titles, and the general topics of justice, diversity, inclusion, vulnerability, marginalization, resiliency, community science, Indigenous knowledge, and related topics appearing in over 200 oral sessions, poster sessions, Town Halls, and other session modalities. The meeting featured a Presidential Forum Lecture by Dr. Bullard and the AGU provided suggested itineraries for attendees centered around themes such as “Community Science” and “Diversity, Equity, and Inclusion.” The annual theme selected for AGU FM21 was “Science is Society”—with the refrain of “We Have to Act Now” to address environmental and social issues proclaimed by AGU Chief Executive Officer Randy Fiser.

Many of the 2021 Fall Meeting sessions highlighted the benefits of including community voices, which results in positive community impacts or equitable policy changes informed by scientific research, clearly demonstrating the ability of AGU scientists and practitioners to heed the call of “We Have to Act Now.” However, several authors of this commentary, as well as presenting authors at FM21 in environmental justice‐adjacent divisions, expressed a lack of preparation and training for achieving equitable research. Training in this form of research is not widely prevalent in geoscience education, with less than 30% of Geoscience faculty teaching Environmental Justice issues (Beane et al., 2019). While basic research remains vitally important to the production of knowledge, many AGU sections, particularly GeoHealth, are both well‐positioned and ethically obligated to conduct science that can directly improve lives. Many of the Environmental Justice sessions at AGU FM21 offered best practices for scientists, academics, practitioners, and funding agencies in GeoHealth and beyond to co‐create solutions for maximal societal benefit.

## 3 Suggested Actions

The following sections contain examples and guidance on Community Science and Trust Building (Section 3.1), Restructuring of Scientific Agency Priorities and Career Expectations (Section 3.2), Opportunities in Education (Section 3.3), and Raising Awareness and Advocating for Policy Change (Section 3.4). While we provide suggested actions for individual researchers, the bulk of our work addresses the necessary actions that must be taken at the institutional level. Institutional action is required to remove the barriers to quality community‐engaged research that many practitioners face due to the inequitable systems perpetuated throughout the scientific enterprise. The suggested actions are summarized in Table 1 but elaborated and emphasized in the subsequent sections.

**Table 1:** Summary of Suggested Actions and Recommendations for Individual Practitioners, Professional Scientific Societies, Funding Agencies, and Research Institutions

	Individual practitioners	Professional scientific societies (e.g., AGU)	Funding agencies (e.g., NSF, NASA)	Research institutions (e.g., academia, national research labs)
Community Science and Trust Building	Avoid helicopter research.	Create brokered research or matchmaking programs.	Create brokered research or matchmaking programs.	Create brokered research or matchmaking programs.
	Anticipate the time and space needed to develop trust within communities.		Allow for salary support and other funding for international collaborators, as well as collaborators within domestic communities and civil society organizations.	
	Attain professional development or connect with brokered research or matchmaking opportunities. Be willing to use your research skills in ways that may be separate from your dominant field.			
	Be transparent in your expectations for publishing and capabilities.		Require community letters of support, or community‐based co‐investigators, in proposals.	
	Ensure research outcomes are collaboratively decided upon for community benefit (do not rely on publications and conference presentations).		Allow for outcomes of the research that are not publication‐based only.	
	Toward the end of a collaborative project, research results should be reviewed relative to the original consent process and promised community benefits.		Consider funding extension mechanisms that allow for reiterative processes to ensure community goals are actualized.	
	When seeking consent, accept no for an answer.		Do not fund research that does not have community consent.	
Scientific Agency Priorities and Career Expectations	Consider a two‐speed research program (wherein short‐term research outputs are completed in parallel to more long‐term, thoughtful, and complex research), but be wary of burnout with this method.	Value methods and outcomes of scholarship beyond publications and presentations.	Value methods and outcomes of scholarship beyond publications and presentations.	Value methods and outcomes of scholarship beyond publications and presentations.
	Advocate in your institutions for policies that recognize the breadth of research outcomes.		Develop strategies designed to fund longer timelines and extensions.	Create tenure and promotion criteria that value quality and impact over quantity.
	Even if your career has been limited by tenure and promotion systems that value single‐author, small team, and fast‐paced research projects, consider the benefits to your research, institution, and junior colleagues when you support promotions that reward broad disciplinary teamwork, justice‐oriented collaborations, and quality over quantity.		Prioritize research proposals with robust and sustainable equity‐driven broader impacts.
			Create frameworks that remove ambiguity in reviewer evaluations of broader impacts proposals.	
			Require a portion of grant money to be set aside for impact evaluation.	
			Require confirmation that funded research has met community expectations, and avoid renewing funding for projects or research groups that over‐promise and under‐deliver on work with historically marginalized communities.	
Education, and	Ensure material that you teach are inclusive, feature active learning that promote success for all students and is justice‐oriented.	Develop learning outcomes, curriculum, and educational requirements that faculty and schools can look to for guidance on supporting student learning from a justice framework.	Measure and evaluate environmental justice as a requirement of funding claiming to establish educational broader impacts.	Develop learning outcomes, curriculum, and educational requirements support student learning from a justice framework.
	Seek educational resources, such as Environmental Data‐Driven Inquiry and Exploration (EDDIE), that help students to connect science to their local communities and/or general public.			
Awareness and Policy	Advocate for Environmental Justice topics at professional society meetings and within policy initiatives.	Support the creation of Policy Committees in order to become active and involved with broader policy initiatives.		
	Attend and be present at sessions highlighting environmental justice issues.	Create a permanent Environmental Justice track, theme, or section at scientific meetings.		
	Vote for and support policy‐makers whose platforms recognize and work to solve environmental injustices.	Implement and evaluate inclusivity and competitiveness criteria for recruiting nominees and conducting society elections and appointment processes.		

### 3.1 Community Science and Trust Building

Working directly with impacted communities and building trust is a critical step to ensuring co‐created solutions. Examples of successful community science collaborations were presented across FM21, especially in talks that highlighted the work of AGU's Thriving Earth Exchange. Thriving Earth Exchange is an AGU‐sponsored program that connects communities with scientists. The overarching theme of the talks can be summarized by the words of Julia Kumari Drapkin, founder of ISeeChange, “you can't solve for geophysical risks withIN communities withOUT communities” (Goodwin, 2021). Partnerships like Thriving Earth Exchange recognize this fact and allow for the community itself to be the leader in these projects. The communities identify the issue and seek scientific input—thereby disrupting a common inequitable form of research, such as “helicopter research” (Haelewaters et al., 2021) or “parachute research” (Hayhow et al., 2021).

Helicopter research arises when privileged scientists travel to marginalized areas to collect data, and then process, analyze, and publish results with little to no involvement from local collaborators (Haelewaters et al., 2021). Within the United States, international research efforts often fall into helicopter research territory as a symptom of US federal funding limitations. For example, the National Science Foundation (NSF) and the National Aeronautics and Space Administration (NASA) do not allow funding to support non‐US researchers and collaborators. The National Institutes of Health (NIH) does allow for salary support of local investigators, though issues of national security in the context of global research collaborations have recently made international collaborations more complex (Redden, 2019). While these limitations do not necessarily preclude paying local US‐based collaborators, they contribute to a system that makes conducting helicopter science advantageous to research careers (see Section 3.2).

Many sessions and presentations reiterated the inadequacy of helicopter research and emphasized not only acknowledging, but actively prioritizing that community members are experts within their communities through the implementation of research methods that are co‐developed with those impacted, ultimately leading to better and more impactful scholarship (Bullard, 2021; Carrasquillo, 2020; Carter et al., 2021; Dhaliwal et al., 2021; Hoffman‐Hall et al., 2021; Martinez et al., 2021; McAdams & White, 2021). An important caveat to this, however, is that any area that has experienced environmental injustice likely also suffers from a long history of helicopter researchers stealing ideas and misusing information with inadequate acknowledgment, overpromises, and false promises.

The legacy of decisions made years (sometimes centuries) ago have lasting impacts on health today. Policies such as redlining and the placement of solid waste and other environmentally detrimental facilities in historically marginalized communities have eroded the trust that many communities have in government, medical, and scientific agencies. Helicopter research can further exacerbate distrust. During the AGU FM21 Town Hall event “Surviving Global Change: GeoHealth, Marginalized Communities, and Environmental Justice in the Anthropocene” (Hoffman‐Hall et al., 2021) Dr. Darryl Reano provided examples of scientists and practitioners who have bypassed Tribal Governments when attempting to collaborate with Indigenous communities. They cautioned that gaining consent from a single member of an Indigenous community does not constitute consent from the community as a whole (Tsosie et al., 2019). Additionally, they urged funding agencies to request letters of support from community leadership or other proof that grant awardees are equitably engaged in trusting relationships with Indigenous communities before, during, and after the proposal submission process (Tachera, 2021).

Going further than letters of support, we also advocate for funding agencies to invest in procedures to internally document the consent processes of local, state, and Tribal governments. With this knowledge, proposal reviews and grant managers can cross‐check if a proposal aligns with policy. This structure gives funding agencies leadership responsibility for educating their funded communities with evolving policies to ensure equity, in addition to legal and ethical compliance, without placing the burden of standard setting on marginalized communities or individual scientists.

Building relationships with communities takes time and trust. A barrier to community science encountered specifically by early career researchers is difficulty in knowing where to begin. The AGU Leadership Academy and Network for Diversity and Inclusion in the Geosciences (AGU LANDInG) offers a range of trainings and webinars aimed at addressing this barrier. For example, in a recent webinar a simple starting point to community science was suggested—showing up to community meetings and listening (Pandya, 2022). Additionally, “brokered research” is a mechanism that connects researchers and communities, whereby large scientific institutions broker community science connections through a match‐making process (Hayhow et al., 2021). Funding agencies, scientific organizations, and academic and research institutions are well positioned to create opportunities to connect communities with researchers. A successful example of this is the AGU Thriving Earth Exchange, which has created meaningful and productive collaborations through brokered research (Faust & Esposito, 2021; Goodwin, 2021; Hammock et al., 2021; Harris et al., 2021; Jones, 2021; Pandya et al., 2014). Individuals and practitioners can and should avail themselves to being matched in this way, though, particularly for early career researchers, imposter syndrome can limit their participation if community needs are not an “exact fit” for their research specialty. Being willing to engage in community‐led and prioritized research can look like traditional research, but also requires research‐adjacent skills that nearly all early career scientists are well positioned to provide regardless of field, such as project management, networking, and scientific communication.

Building a non‐exploitative and positive relationship takes both patience and transparency—it is important when first interacting with a community to be transparent about the expected outcomes of the research, such as publications or presentations, to avoid creating an exploitative relationship. It is also important to ensure that publications or conference presentations are not the *only* outcomes of the work, as traditional academic publications and conferences are often available only behind expensive paywalls or registration fees. Additionally, toward the end of a collaborative project, research results should be reviewed relative to the originally promised community benefits. Questions to ask at the end of a project include, what did the community expect and agree to participate in (conversely, what did they not agree to or want to avoid)? What did the community (not just the scientists) learn through the research project? Did the project provide the stated benefits intended? If the answer to any of these questions is no, additional iterations of the work will be necessary, with implications for funding agency timelines (see Section 3.2 for more).

Recent work by Filippelli et al. (2021) demonstrates the importance of community trust and empowering and involving communities in the development of parallel information products that are designed with and for the general public. Through a partnership between Indiana University (IUPUI) and local Indianapolis faith‐based organizations, residents were provided with tools to assess lead exposure in their households anonymously. The anonymity aspect was significant as some community members were hesitant to pursue professional lead testing due to concerns about government repercussions. Participants benefit directly from the testing and information provided by the researchers at IUPUI while the community as a whole benefits from the data being anonymously integrated into a public‐facing Map My Environment website (https://www.mapmyenvironment.com/).

Lastly, and most importantly, it is imperative to be willing to accept “no” for an answer and to *never* *fund* *or conduct research with the stated purpose of community benefit, without community consent*.

#### 3.1.1 Suggested Actions

Individual Scientists/Practitioners

- Avoid helicopter research.
- Anticipate the time and space needed to develop trust within communities.
- Attain professional development or connect with brokered research or matchmaking opportunities. Be willing to use your research skills in ways that may be separate from your dominant field.
- Be transparent in your expectations for publishing and capabilities.
- Ensure research outcomes are collaboratively decided upon for community benefit (do not rely on publications and conference presentations).
- Toward the end of a collaborative project, research results should be reviewed relative to the original consent process and promised community benefits.
- When seeking consent, accept no for an answer.

Professional Scientific Societies

- Create brokered research or matchmaking programs.

Funding Agencies

- Create brokered research or matchmaking programs.
- Allow for salary support and other funding for international collaborators, as well as collaborators within domestic communities and civil society organizations.
- Require community letters of support, or community‐based co‐investigators, in proposals.
- Allow for outcomes of the research that are not publication‐based only.
- Consider funding extension mechanisms that allow for reiterative processes to ensure community goals are actualized.
- Do not fund research that does not have community consent.

Research Institutions

- Create brokered research or matchmaking programs.

### 3.2 Restructuring of Scientific Agency Priorities and Career Expectations

One of the greatest barriers to building relationships and trust between scientists/practitioners and communities is the misalignment of expectations over the time horizon of communities experiencing injustice and academic processes. In a review of participatory research for environmental justice from 1992 to 2020, Davis and Ramirez‐Andreotta (2021) found that research leading to structural change is more likely when projects are long‐term (>4 years). They note, however, that this is usually only possible with multiple funding mechanisms, due in part to the compressed timelines provided by many research grants. Additionally, engaging in community‐led research requires careful (and therefore sometimes, slow) scholarship that is worth less than fast‐paced scholarship in the neoliberal university (Mountz et al., 2015).

While many early career AGU researchers at FM21 professed interest in co‐producing research and solutions with local communities, they discussed having limited support in building lasting and meaningful relationships due to time‐to‐degree, time‐to‐tenure, grant deliverables deadlines, or other career expectations. As noted by Cannon (2020), rationally, junior faculty and doctoral candidates should conduct secondary data analysis on already existing datasets, in order to publish as quickly as possible, given the hyper‐competitive academic job market. It follows then, that junior faculty who engage in community‐driven research are working against their own self‐interests (Cannon, 2020). For individual early stage academics, a possible solution is a two‐speed research program McCabe, 2012, wherein short‐term research outputs are completed in parallel to more long‐term, thoughtful, and complex research (the authors of this paper do not necessarily promote this solution, which can lead to burnout and work‐life imbalances, but will likely be viewed more favorably in tenure and promotion decisions).

For academic institutions, addressing time‐to‐degree and time‐to‐tenure restraints can often be “solved” through time extensions, however, it has been well‐documented that time extension policies often exacerbate inequities in academia (Antecol et al., 2018; Malisch et al., 2020). Instead, recommendations for academic institutions can be found in Indigenous, disability, and feminist scholarship ‐ some of which offer complete reimaginings of the academy away from hyperproductive and hypercompetitive institutions to places where colleagues are collaboratively responsible for tenure/graduation, thereby creating a tenure process that is not akin to only the most exceptional scholars moving past a gatekeeper, but rather, an expected move into a secure position (Medak‐Saltzman et al., 2022). However, as described by Carson et al. (2019) shifting the longstanding academic culture that values grant funding and publications above all will take a considerable amount of time to achieve. In the meantime, smaller incremental steps can be taken to move the academy toward valuing the time it takes to do something well (as is necessary when co‐producing with communities), rather than the quantity of work that one can produce in a certain time frame.

For example, in academic institutions and fields where single‐authored work is more highly valued for tenure and promotion, collaborative scholarship that benefits from diverse teams and broad disciplinary input will be diminished. Health disparity research in particular benefits from community engagement and collaborations between scholars from different disciplines (Cannon, 2020). Co‐authorship from those outside of the traditional scientific research space should be seen as similarly valued, if not outright required, for scientists working within historically marginalized communities. Tenure and promotion policies can also be reworked to place similar weight to scholarship activities beyond grant funding and publications. Research outputs such as structural benefits to marginalized communities, articles in popular media, lectures to non‐scientific audiences, and more have often been used as an additional metric of assessment for tenure track scholars, as opposed to equally worthy endeavors.

Similarly, multiple panel sessions at AGU FM21 emphasized the need to restructure funding agency priorities, particularly a de‐emphasis on deliverables such as publications, reports, and presentations and a greater emphasis on impacts and partnerships. Specific to the GeoHealth section's mission is the National Science Foundation (NSF) commitment to Broader Impacts (BIs), one of which is the “improved well‐being of individuals in society.” In health research, it is often taken for granted that well‐being will be improved by the nature of the work; however, it is important that funding agencies consider the robustness of proposed BI in terms of their ability to advance equity and remain sustainable beyond the life of the grant. While the frameworks for assessing BI at the proposal stage aim to do this, many have identified areas in which these frameworks fall short or otherwise leave too much ambiguity in their influence on the funding process (Bozeman, 2020; Sarewitz, 2011; Watts et al., 2015; Woodson & Boutilier, 2022). Further, funders need to consider the implementation of promised BI activities. Particularly for community‐engaged research, to promise a broader impact and receive funding to make that a reality, and then fail to make a good‐faith effort to create that impact, at best represents a planning failure that should preclude the researcher from further funding, or at worst represents reprehensible ethics. Bozeman (2020) proposes a solution to this, calling for grants to require some percentage of funding set aside for impact evaluation. While this is rare across US federal funding agencies, it is not completely radical—for example, NSF already requires money to be set aside for external evaluation for proposals to its ADVANCE program for improving gender equity in science.

#### 3.2.1 Suggested Actions

Individual Scientists/Practitioners

- Consider a two‐speed research program (wherein short‐term research outputs are completed in parallel to more long‐term, thoughtful, and complex research), but be wary of burnout with this method.
- Advocate in your institutions for policies that recognize the breadth of research outcomes.
- Even if your career has been limited by tenure and promotion systems that value single‐author, small team, and fast‐paced research projects, consider the benefits to your research, institution, and junior colleagues when you support promotions that reward broad disciplinary teamwork, justice‐oriented collaborations, and quality over quantity.

Professional Scientific Societies

- Value methods and outcomes of scholarship beyond publications and presentations.

Funding Agencies

- Value methods and outcomes of scholarship beyond publications and presentations.
- Develop strategies designed to fund longer timelines and extensions.
- Prioritize research proposals with robust and sustainable equity‐driven broader impacts.
- Create frameworks that remove ambiguity in reviewer evaluations of broader impacts proposals.
- Require a portion of grant money to be set aside for impact evaluation.
- Require confirmation that funded research has met community expectations, and avoid renewing funding for projects or research groups that over‐promise and under‐deliver on work with historically marginalized communities.

Research Institutions

- Value methods and outcomes of scholarship beyond publications and presentations.
- Create tenure and promotion criteria that value quality and impact over quantity.

### 3.3 Opportunities in Education

Many discussions at AGU FM21 highlighted a distinct lack of Environmental Justice topics and training within scientific education, particularly how to connect science to community needs. While a common conversation of educating graduate students is a great start, there is also a need to educate across the entire career spectrum, from K‐12 to more senior researchers and academics, on environmental ethics and prioritizing community expertise when researching or working in historically marginalized areas. Additionally, informal education within communities, through outreach, collaboration, and citizen science models has been well‐informed through frameworks developed by groups like AGU's Thriving Earth Exchange. However, the undergraduate educational experience represents an opportunity for growth.

Educating undergraduates is particularly impactful since they will take these concepts into science and other professions throughout their careers, all of which benefit from strategies for equitable, inclusive, and creative approaches to work for change. Undergraduate education influences a student's social agency, namely, their ability to influence or transform society through activities like civic engagement or community organizing (Aoki et al., 2022). However, research has shown that STEM students typically believe that working for social change is less important to their career goals than non‐STEM students, which persists across their path to graduation (Garibay, 2015). Integrating Environmental Justice into undergraduate education (particularly within STEM departments) is valuable for encouraging students to think critically about scientific paradigms, which are still presented from a largely euro‐centric viewpoint (Milne, 2011), and ultimately increase their social agency.

At the undergraduate level, the AGU FM21 workshop Building Quantitative Literacy Through Science, Education, and Art showcased the ready‐made teaching modules of Project Environmental Data‐Driven Inquiry and Exploration (EDDIE) (Fortner, Mode, et al., 2021; Fortner, Suffolet, et al., 2021). Spanning a broad range of environmental topics, EDDIE modules support student quantitative literacy through scaffolding quantitative reasoning skills and building student expertise with the nuances of Earth and environmental analyses (O'reilly et al., 2017). What connects EDDIE to Environmental Justice is that the real‐world focus of modules offers the opportunity for synergies through art that enhance student learning and help students take the science they have learned and co‐create ideas with broader audiences. For example, during the workshop Artist Jiabao Li presented Glacier's Lament, which features a cellist playing an increasingly urgent and haunting musical piece tracking the retreat of the Mendenhall Glacier. The artistic methods displayed during the workshop deepen learning and connection to science and broaden participation needed to support social change by building student capacity to translate scientific findings to public audiences in unique ways.

Fortner, Mode, et al. (2021) and Fortner, Suffolet, et al. (2021) experimented with incorporating Environmental Justice and co‐creating solutions into an undergraduate environmental science course. For the course, students met with a local community partner who communicated the desire to create community gardens as a way to combat food deserts. The area has several vacant lots that could be used for this purpose. The students then developed a sampling plan and campaign, where they measured the organic carbon and lead content of the soils. Relating their findings to historic racist housing practices by the Home Owners' Loan Corporation (HOLC) (redlining), the students were able to suggest the best lots to plant community gardens.

To increase the exposure of students to Environmental Justice issues, Dhaliwal et al. (2021) presented a workshop series in which undergraduates were encouraged to discuss the ethics of scientific decisions, with the guidance of graduate and postdoctoral mentors. To provide a framework, students were assigned books that explored the themes of extractivism on Indigenous lands and inclusivity in science. In these discussions, undergraduates readily engaged in the subject matter, made a notable effort to understand and connect to the sometimes difficult or unfamiliar themes, and lamented that more of their faculty and instructors did not discuss Environmental Justice issues as openly as within this workshop series. This work demonstrates that while undergraduates are willing and interested in Environmental Justice, more opportunities need to be developed for them to engage with this concept.

The strategies and work presented here can be useful in determining and defining the goals of any proposed research with broader impacts that have a justice‐oriented undergraduate education focus. Specifically, projects can aim to: build student expertise in the nuances of environmental analysis, cultivate opportunities to co‐create ideas with broader audiences, incorporate real‐world modules, highlight synergies with art, build student capacity to translate scientific findings, discuss the ethics of scientific decisions, explore and engage with unfamiliar themes in science (e.g., extractivism, inclusivity, indigenous justice), and connect scientific ideas to students' personal identities and communities.

#### 3.3.1 Suggested Actions

Individual Scientists/Practitioners

- Ensure material that you teach are inclusive, feature active learning that promote success for all students and is justice‐oriented.
- Seek educational resources, such as EDDIE, that help students to connect science to their local communities and/or general public.

Professional Scientific Societies

- Develop learning outcomes, curriculum, and educational requirements that faculty and schools can look to for guidance on supporting student learning from a justice framework.

Funding Agencies

- Measure and evaluate environmental justice as a requirement of funding claiming to establish educational broader impacts.

Research Institutions

- Develop learning outcomes, curriculum, and educational requirements support student learning from a justice framework.

### 3.4 Raising Awareness and Advocating for Policy Change

The Environmental Justice themes at AGU21 created a strong call for Environmental Justice to continue to be a major fall meeting topic each year. Much like infectious diseases, wildfires, and air quality are consistently well represented among GeoHealth sessions, Environmental Justice should have a permanent presence at Fall Meetings across all sections. Environmental Justice, a field that engages many disciplines, has a history of impactful and transformative scholarship which will enhance the Fall Meeting experience. Additionally, many of the scholars of Environmental Justice are members of marginalized communities and thus could provide valuable and sorely needed insights given the AGU's lack of diverse membership (Fiser, 2021).

The AGU Fall Meeting 2022 (FM22) is shaping up to include high‐priority Environmental Justice topics across disciplines and will offer learning and sharing opportunities focused on building capacity and training for researchers. Example proposed sessions from the GeoHealth and Science & Society sections include “Bringing Health to the Room Where it Happens: Incorporating Health and Equity into Climate Policies and Planning,” “Geospatial Data Applications for Environmental Justice,” and “Advancing Justice and Equity Through Co‐produced Research.” Additionally, AGU FM22 will have more workshop and training opportunities for researchers, such as “Establishing Collaborative Partnerships for Equity and Justice” which will be organized by the GeoHealth Policy Committee and will feature community collaborators and Environmental Justice practitioners.

Beyond Fall Meeting, AGU members share a heightened level of privilege that creates a moral obligation to advocate for continued Environmental Justice advocacy and research within broader policy spaces. New federal initiatives, like the United States Department of Health and Human Services (HHS) Office of Climate Change and Health Equity, the White House Justice40 Initiative, and the White House Environmental Justice Advisory Council allow for opportunities for AGU members and leadership to coordinate in meaningful ways toward action on Environmental Justice issues and community capacity building. While GeoHealth and other AGU research consistently demonstrate environmental injustices, the high partisanship surrounding racial and social justice issues (Pew Research Center, 2021) increases the urgency with which these broad policy initiatives need to be elevated.

The GeoHealth section has created a specific Policy Committee that works closely with AGU staff in order to encourage policy actions taken by the Section or by members of the Section, and encourage their incorporation into public policy at local, state, national, and international levels. Recent successes of the GeoHealth Policy Committee include collaborating with AGU on their endorsement of the Comprehensive National Mercury Monitoring Act, as well as a recommendation letter to the US Office of Science and Technology Policy in favor of rural climate change priorities for federal activities, to include: improving rural‐urban coordination, investment in climate resilience projects for rural communities, engaging rural stakeholders in climate solutions, emphasizing equity for rural communities in health outcomes, and expanding evaluation of climate initiatives across the rural‐urban landscape. To our knowledge, very few of the other AGU Sections have dedicated Policy Committees. We encourage each section to consider creating their own.

Lastly, nearly all of the actions suggested throughout this work require strong, inclusive, and diverse leadership. At the institutional level, this means implementing and evaluating for inclusivity within the criteria for recruiting nominees within society elections and appointments. Similarly, for individuals, exercising your right to vote, advocating for the rights of others to vote, and supporting policy makers whose platforms recognize and work to solve environmental injustices are important and meaningful ways to advance Environmental Justice broadly across society.

#### 3.4.1 Suggested Actions

Individual Scientists/Practitioners

- Advocate for Environmental Justice topics at professional society meetings and within policy initiatives.
- Attend and be present at sessions highlighting environmental justice issues.
- Vote for and support policy‐makers whose platforms recognize and work to solve environmental injustices.

Professional Scientific Societies

- Support the creation of Policy Committees in order to become active and involved with broader policy initiatives.
- Create a permanent Environmental Justice track, theme, or section at scientific meetings.
- Implement and evaluate inclusivity and competitiveness criteria for recruiting nominees and conducting society elections and appointment processes.

## 4 Conclusions

Environmental Justice principles are an essential foundation for GeoHealth research and action. AGU and its 130,000 worldwide members can play a key role in creating change for historically marginalized communities. However, the current structures of academia and scientific research create significant challenges to individual researchers working to co‐create just solutions to environmental injustices. The suggested actions for institutions and organizations presented here represent the most critical aspect of this work. Many of the barriers to community‐engaged scholarship that individual scientists face are not self‐imposed, but symptoms of a scientific system built on a foundation of white supremacy. While we applaud the meaningful steps that organizations like AGU and NSF have taken to address historical wrongs, the urgency in taking the actions proposed here cannot be understated. For funding agencies, funding mechanisms that ensure community consent, provide for extensions and reiterative/evaluative processes, value research outputs beyond publications, and prioritize robust broader impacts (and reject those that over‐promise and under‐deliver) are long‐overdue and critical to creating just solutions within historically marginalized communities. For academic and research institutions, a large‐scale overhaul of tenure and promotion criteria is necessary, especially to engage more early career researchers in this work. Finally, for organizations that truly value Environmental Justice, the creation of brokered research programs and justice‐orientation curricula are extremely valuable and transformational inputs to the academic system.

While some of the suggested actions for organizations may take substantial time and investment, many of the actions provided for individuals can be taken more immediately. We recognize that many researchers have been constrained by career expectations, inequitable funding mechanisms, or other structural issues inherent in the current research system. However, even small steps toward a justice‐oriented practice can create disruptions in the injustice experienced by marginalized communities. In your research, if you are new to community science, consider connecting with brokered research organizations, be transparent in your expectations and capabilities, and attend scientific sessions that highlight this important work. Advocate for institutional policies that value the breadth of research outcomes beyond publications and, when teaching, do so with a justice‐oriented framework that allows students to connect their ideas to their communities.

Overall, including historically marginalized communities in geoscience research results in higher quality research and broad, critical, and timely societal impacts. We hope that the recommendations provided in this paper can serve as a catalyst for physical scientists, social scientists, educators, funding agencies, academic institutions, and the leadership of AGU and other scientific societies to engage more fully with the communities they serve to alleviate inequities in a way that prioritizes the rights, sovereignty, knowledge, and dignity of those communities.

## Conflict of Interest

The authors declare no conflicts of interest relevant to this study.

## Data Availability

All sessions described are available in the AGU Fall Meeting 2021 Program: https://agu.confex.com/agu/fm21/meetingapp.cgi/Home/0.
